# Symptomatic Emotional Responses and Changes in Networks Elicited by Direct Electrical Stimulation

**DOI:** 10.1111/cns.70393

**Published:** 2025-04-17

**Authors:** Menglin Liu, Ying Gao, Guiliang Hao, Xiaoming Yan, Xiaohua Zhang, Xueyuan Wang, Wei Shu, Tao Yu

**Affiliations:** ^1^ Beijing Institute of Functional Neurosurgery Xuanwu Hospital, Capital Medical University Beijing China

**Keywords:** direct electrical stimulation, emotion, emotional responses, epilepsy, stereoelectroencephalography

## Abstract

**Aim:**

Emotion is a major area of research in psychology and neuroscience. However, the role of direct electrical stimulation (DES) in emotional localization has not yet been fully explored. This study aimed to analyze the use of DES in examining the local connectivity of brain regions eliciting emotional responses, thereby providing evidence for a new perspective of local changes in brain networks during emotional responses.

**Methods:**

We reviewed the clinical data of 500 patients with refractory epilepsy who underwent stereoencephalogram (SEEG) implantation to locate the epileptogenic area and functional mapping of the brain. The three‐dimensional reconstruction was employed for the qualitative and positioning analysis on the emotional responses elicited using DES. We used Granger causality (GC), directed transfer function (DTF), and partial directed coherence (PDC) to analyze the changes in functional connectivity before and after stimulation in selected patients.

**Results:**

Emotional responses were evoked without aura using DES in 85 contacts in 31 patients, including 35 (41.2%) contacts with fear, 37 (43.5%) contacts with happiness, 6 (7.1%) contacts with anxiety, and 7 (8.2%) contacts with depression. Three contacts of interest in two patients experiencing transient emotional symptoms underwent GC, DTF, and PDC analyses; the analysis revealed significant differences in brain networks before and after stimulation in selected patients.

**Conclusions:**

DES can evoke emotions across various brain regions, such as the bilateral amygdala, hippocampus, temporal lobe, frontal lobe, insula, cingulate cortex, paracentral gyrus, fusiform, orbitofrontal cortex, left thalamus, and putamen. These elicited emotional experiences may largely result from the alterations in local brain networks.

## Graphical abstract

This study used electrical stimulation of both cortical and subcortical structures in patients with implanted SEEG electrodes. Mapping found that emotional responses are widely elicited by multiple cortical regions and subcortical nuclei. Further local network analysis revealed that the elicitation of emotional responses depends on changes in local networks rather than being attributed to single cortical functions.

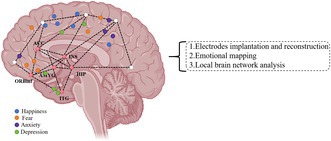

**Abbreviations**
ACC: anterior cingulate cortex
AMYG: amygdala
CAU: caudate nucleus
FFG: fusiform gyrus
HIP: hippocampus
INS: insula
ITG: inferior temporal gyrus
LOC: lateral occipital cortex
MFG: middle frontal gyrus
MTG: middle temporal gyrus
OFC: orbitofrontal cortex
PCL: paracentral lobule
PCUN: precuneus
PreCG: precentral gyrus
ProCG: percentral gyrus
PUT: putamen
SFG: superior frontal gyrus
SMG: supramarginal gyrus
STG: superior temporal gyrus
THA: thalamus

## 1 Introduction

The creation of emotions by the brain has been a fundamental area of research in psychological science. Two predominant schools of thought have been proposed to understand emotions in the human brain: the locationist hypothesis and the psychological constructionist hypothesis. The locationist approach hypothesizes that discrete categories of emotion correspond to specific and consistent brain regions [1], assuming that emotional responses (disgust, anger, happiness, fear, and sadness) are elicited by activities within an architecturally defined brain locale or anatomically defined networks of several locales [2, 3, 4, 5]. In contrast, the psychological constructionist hypothesis states that discrete emotions are constructed from more general networks in the brain not specific to dedicated anatomical regions in the brain [1]. A psychological constructionist assumes that emotions represent psychological events emerging from simple psychological events that do not specifically relate to emotion [1]. In this context, some emotional categories are not limited by the human brain, including sadness, anger, and fear [6, 7, 8]. Instead, this approach assumes that the psychological functionality of distinct regions in the brain is determined by a network of brain regions, at least in part [9]; hence, psychological events are generated by the review of large‐scale brain networks [10, 11, 12, 13]. Core affect is a term used in the psychological constructionist hypothesis to describe the fundamental form of sensory inputs received by the body, including raw somatic, visceral, vascular, and motor cues, as well as feelings of arousal or affect [14, 15, 16, 17]. Therefore, core affect is used to describe the mental representation of the changes in the body experienced as pleasure or displeasure with some degree of arousal; these feelings occur in the body but are represented in the brain [14, 15, 17].

Further, other perspectives have been used to investigate the formation of human emotions. For example, Papez proposed a distributed mechanism responsible for emotion, involving structures such as the hypothalamus, hippocampus, anterior thalamus, and cingulate gyri. Subsequent research introduced the region versus circuit tension hypotheses, including MacLean's proposal of the visceral brain or limbic system [18] and Panksepp's framework of specialized subcortical circuits for basic emotions [19]. Pessoa proposed a functional, integrated system featuring a complex network of cortical and subcortical structures collaborating to regulate emotions and associated behaviors [20].

Functional magnetic resonance imaging (fMRI) can detect the changes in blood oxygen level–dependent signals from specific brain regions; these reflect functional changes in these areas. Numerous studies have demonstrated the capability of healthy individuals to regulate their brain activity when exposed to fMRI in different regions associated with emotional regulation, such as the amygdala, anterior insula, and anterior cingulate cortex [21]. Besides conducting imaging studies on brain activity, fMRI can analyze functional connectivity between different brain regions. For example, a previous study demonstrated that fMRI neurofeedback in response to aversion stimuli increased connectivity between the prefrontal lobe and the limbic system, specifically the amygdala [22]. Moreover, another study reported initiating a fear‐related functional network, comprising the amygdala, insula, hippocampus, and anterior cingulate cortex, upon activation of the amygdala [23].

However, despite the many advantages of fMRI, a limitation of this technology is that inherent defects, including low accuracy, non‐individuality, and false positivity, can influence our in‐depth understanding of emotional responses [24].

Direct electrical stimulation (DES) is another technique used to investigate brain function, representing a powerful method for investigating the functional cortex in humans. This technique can provide valuable information related to both the localization of the epileptogenic zone (EZ) and the definition of cortical functionality, and has been used for the cortical mapping of patients with refractory epilepsy for decades [25]. Furthermore, researchers have proven that DES can trigger transient emotional responses [26]. However, systematic research on the focal cortical areas directly related to emotion is still inadequate. Guillory et al. conducted a review of over 60 years of human intracranial electrophysiology data to investigate the basis of emotion [27]. They found that multiple emotional responses could be induced by stimulating multiple cortices, including the amygdala, orbitofrontal cortex, and insula. Furthermore, these cortices may exert functionality for separate emotional responses [28]. The functionality of the cortex in the sulci or deep cortex (cingulate gyrus, insula, amygdala, and hippocampus) could not be investigated in detail due to the limitations of subdural electrodes. However, the increasing popularity of stereoelectroencephalography (SEEG) has overcome this shortcoming in recent years. The corresponding relationship between SEEG electrodes and cortical sites has become increasingly more accurate with the continuous development of MRI technology [29], thus enabling researchers to further complement and improve the functional map of emotions in the human brain.

In the present study, we reviewed DES‐induced data in different brain regions in a relatively large cohort of patients with epilepsy implanted with SEEG electrodes. We investigated single cortical functions and functional connectivity by analyzing the electric brain activities evoked using DES.

## 2 Methods

### 2.1 Patients

We reviewed the data acquired from patients with drug‐resistant epilepsy who underwent SEEG during presurgical evaluation at the Beijing Institute of Functional Neurosurgery between June 2016 and January 2023. We selected patients who received SEEG implantation to localize the EZ. DES was performed after the implantation of electrodes and long‐term video‐EEG monitoring, and patients experiencing emotional responses were included in our analysis. The accuracy of our investigation of physiological response was increased by excluding patients with obvious dysplasia at the neuroanatomical level and those lacking reliable DES results owing to mental or cognitive disorders.

All patients provided informed consent after admission, and legal guardians provided consent for underage participants. Patients exhibited no clinical evidence of mental or cognitive disorders. This study was approved by the Medical Ethics Committee of Xuanwu Hospital, Capital Medical University (Approval reference: LYS [2024] 297‐001).

### 2.2 Presurgical Evaluation

All patients underwent high‐resolution 3.0‐T MRI scans, including spin‐echo T1‐weighted, T2‐weighted, and fluid‐attenuated inversion recovery sequences, as well as three‐dimensional (3D) anatomic T1‐weighted axial, sagittal, and coronal sequences covering the whole brain with a section thickness of 0.8 or 1 mm. In addition, magnetoencephalography and positron emission tomography–computed tomography scanning were performed to localize the EZ for most patients. Routine long‐term scalp video electroencephalography (vEEG) monitoring was performed to record at least three habitual seizures. All clinical and neuroimaging data were analyzed by our group to localize the EZs, and subsequent SEEG implantation was designed for patients as necessary.

### 2.3 Implantation of SEEG Electrodes

A Cosman–Roberts–Wells human body stereotaxic frame (Radionics, USA; 2016–2018) and a Sinovation robotic arm‐assisted system (Sinovation Medical Technology, China; 2019–2023) were used to implant intracranial multiple‐contact SEEG electrodes. Each electrode had 8–16 contacts, depending on its length. The electrode diameter was 0.8 mm, with each contact measuring 1.5 mm in length and 2 mm spacing between neighboring contacts. The number of electrodes and their anatomical targeting were based on the information acquired during noninvasive presurgical evaluation and anatomical hypotheses for each patient. A range of open‐source software and toolboxes were applied, including SPM12 [30], Freesurfer [31], and 3D slicer [32], when postoperative high‐resolution computed tomography images were registered with preoperative high‐resolution MRI images. Intracranial electroencephalography (iEEG) monitoring was performed to further localize the EZ in each patient. The iEEG sampling rate was set as 1024 Hz. The duration of video‐EEG monitoring ranged 3–14 days, capturing at least three habitual seizures per patient.

### 2.4 Direct Electrical Stimulation

Patients underwent DES after or during long‐term iEEG monitoring. Bipolar stimulation was applied with a biphasic wave, with parameters set to a pulse width of 0.2 ms, a frequency of 50 Hz, and a duration of 3 s. Following our clinical protocols and previous publications [33, 34] and to minimize the occurrence of pathological after‐discharges (ADs), our current intensity commenced at 0.5 mA and incrementally increased by 0.1‐mA or 0.2‐mA up to a maximum of 6 mA. For each electrode contact, DES was terminated when the current intensity reached 6 mA or definite aura/seizures were elicited. The emotions experienced in patients due to the stimulus were temporary and did not warrant a clinical diagnosis of mental illness.

### 2.5 Functional Connectivity

**FIGURE 1:**
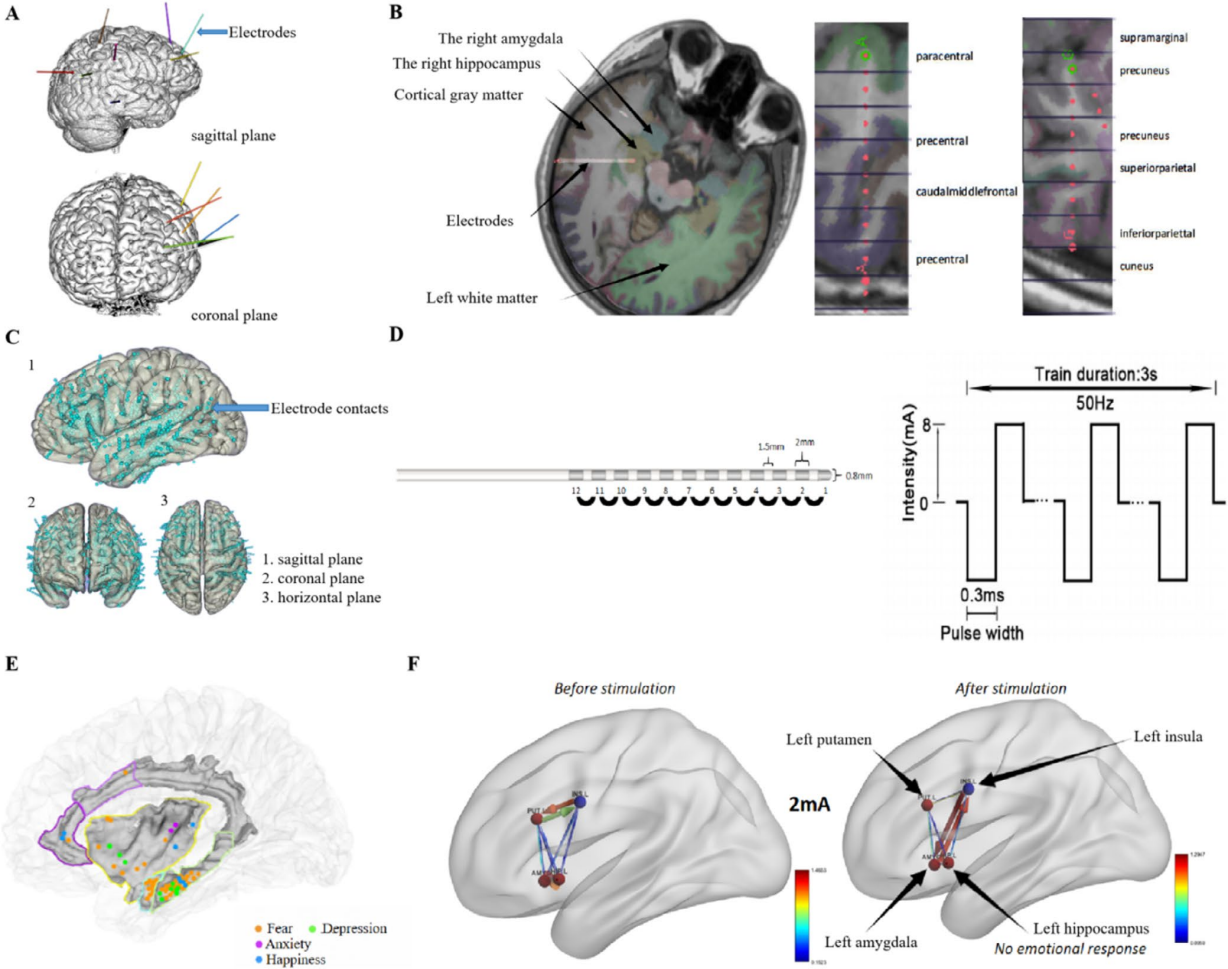
Methods and procedures used in this study. (A) Three‐dimensional (3D) reconstruction of electrode implantation in a patient, generated using 3D slicer software. (B) Computed‐tomography (CT) scan following implantation with preoperative MRI fusion reconstruction, showing electrode position in the brain and gyri for each contact. Red dots represent electrode contacts. (C) Electrode contacts implanted in 30 patients registered on a standard brain map, showing that the electrode contacts of the patients in this study (500 cases) covered almost the entire cortical region. (D) Sample electrode contacts with dimensions and stimulation parameters: electrode diameter 0.8 mm, contact length 2 mm, and 1.5 mm spacing between contacts, stimulation frequency 50 Hz, pulse width 0.3 ms, and amplitude 0–8 mA (square wave stimulation). (E) Schematic diagram showing functional cortical mapping, with each dot representing a contact eliciting an emotional response and different colors indicating different types of emotional responses. (F) Schematic diagram showing the Granger causality (GC) analysis, where dots represent different brain regions, arrows depict functional connections between brain regions, arrow thickness represents relationship strength, and color depth reflects significance level.

We recruited patients who had undergone SEEG implantation in specific brain regions of interest, namely the left amygdala, left hippocampus, and left insula, to better understand the mechanisms responsible for the elicitation of emotion. Then, Granger causality (GC), directed transfer function (DTF), and partial directed coherence (PDC) were used to investigate the changes in the functional connectivity of local brain regions when emotional responses were elicited or not elicited, thus resulting in the generation of visual representations. The data were assessed for normality using theShapiro‐Wilk test. Normally distributed data were analyzed with t‐tests, while non‐normally distributed data were evaluated via the Wilcoxon signed‐rank test to determine the significance of signal intensity so as to verify the significance of changes in functional connection. This helped identify the direction and amount of information flow between different brain regions. Subsequently, the changes in functional connectivities preceding and following emotional responses were examined. We identified internal electrophysiological evidence, than performing systematic electrophysiological analysis of the symptoms, in all patients. The entire methodological process of this study is illustrated in Figure 1.

## 3 Results

This study included 500 patients with refractory epilepsy and approximately 10,000 contacts. Of these, 57 (11.40%) patients with 201 (2.01%) contacts experienced emotional responses. After excluding patients with epileptic aura and AD cases, 29 (5.80%) patients with 85 (0.85%) contacts experienced emotional responses; specifically, fear was elicited by 35 contacts in 16 patients, anxiety was elicited by six contacts in three patients, happiness was elicited by 37 contacts in nine patients, and depression was elicited by seven contacts in three patients. Then, we counted the emotional experiences subjectively experienced by patients during DES. Despite the variations in the subjective descriptions between different patients, these emotional experiences were generally classified into fear, anxiety, happiness, and depression.

### 3.1 Anatomical Localization

**FIGURE 2:**
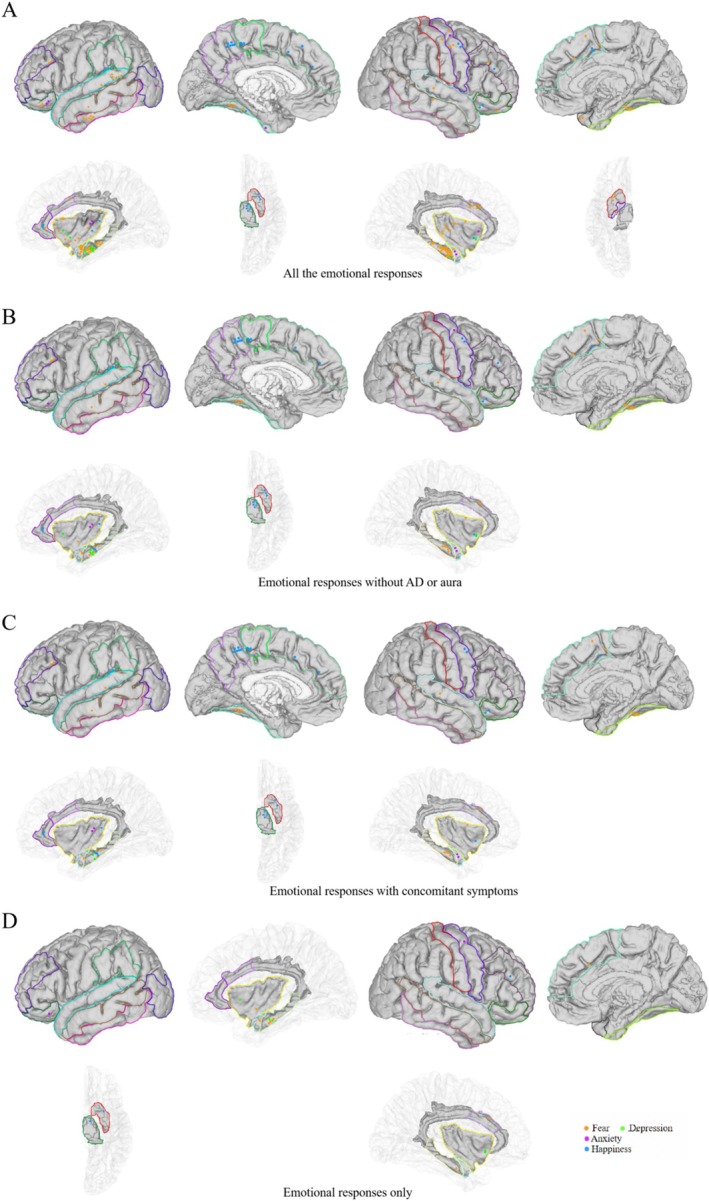
The locations of electrode contacts that evoked positive emotional experiences. The SFG.L, MFG.L, OFC.L, STG.L, MTG.L, ITG.L, SMG.L, PCUN.L, PCL.L, FFG.L, LOC.L, ACC.L, INS.L, AMYG.L, HIP.L, THA.L, PUT.L, PreCG.R, ProCG.R, SFG.R, MFG.R, OFC.R, STG.R, MTG.R, FFG.R, ACC.R, INS.R, AMYG.R, HIP.R, THA.R, PUT.R, and CAU.R. (B) The SFG.L, MFG.L, OFC.L, STG.L, MTG.L, SMG.L, PCUN.L, PCL.L, FFG.L, ACC.L, INS.L, AMYG.L, HIP.L, THA.L, PUT.L, PreCG.R, SFG.R, MFG.R, OFC.R, STG.R, FFG.R, ACC.R, INS.R, AMYG.R, HIP.R. (C) The SFG.L, MFG.L, OFC.L, STG.L, MTG.L, SMG.L, PCUN.L, PCL.L, FFG.L, ACC.L, INS.L, AMYG.L, HIP.L, THA.L, PUT.L, PreCG.R, SFG.R, OFC.R, STG.R, FFG.R, ACC.R, INS.R, AMYG.R, HIP.R. (D) The OFC.L, INS.L, AMYG.L, HIP.L, THA.L, PUT.L, PreCG.R, SFG.R, MFG.R, ACC.R, INS.R, HIP.R. Orange represents fear, purple represents anxiety, blue represents happiness, and green represents depression.

The positive contacts were generally associated with regions including the bilateral hippocampus, fusiform, frontal cortex, anterior cingulate cortex, temporal cortex, insula, amygdala, orbitofrontal cortex, and right anterior central cortex, left paracentral lobule, left dorsal thalamus, left putamen, left precuneus, and left supramarginal cortex. The standard brain map produced by the 3D slicer was used to represent all 112 brain regions in this study. All positive contacts, including auras and AD, are shown in Figure 2A, whereas those without auras or AD are shown in Figure 2B. Considering the potential influence of auras and AD on our analysis, the study focused specifically on patients and contacts shown in Figure 2B and Table 1. Of the 29 patients reporting transient emotional symptoms, 16 reported fear, nine reported happiness, three reported anxiety, and three reported depression. One patient experienced both fear and depression, and one patient experienced both fear and happiness.

**TABLE 1:** Site of electrical stimulation in each patient, stimulation parameters, and categories of emotional experience.

Patient	Location	Current intensity, mA	Emotional symptom
1	Right FFG	1.5	Fear
	Right FFG	2	Fear
	Left FFG	1.5	Fear
	Left HIP	1	Fear
	Left HIP	2	Fear
	Right INS	2.5	Depression
	Right INS	4.5	Depression
2	Right INS	2	Fear
	Right SFG	5	Fear
3	Left INS	4	Fear
	Left HIP	2	Fear
	Left HIP	3	Fear
4	Right ACC	5	Fear
5	Left MTG	2	Fear
6	Left AMYG	3	Fear
7	Left HIP	5.5	Fear
8	Right STG	2	Fear
9	Right HIP	0.8	Fear
	Right HIP	1.5	Fear
10	Right HIP	1.5	Fear
11	Right ACC	2	Fear
12	Left MFG	1.4	Fear
	Left MFG	2	Fear
13	Right SFG	0.5	Fear
14	Left STG	1	Fear
	Left STG	3	Fear
15	Right HIP	1.6	Fear
16	Right SFG	6	Fear
	Right OFC	3	Happiness
	Right INS	4	Happiness
	Right SFG	4	Happiness
	Right OFC	5	Happiness
	Right MFG	6	Happiness
17	Left OFC	3	Anxiety
18	Left INS	2.5	Anxiety
	Left INS	3.5	Anxiety
19	Right AMYG	3	Anxiety
	Right PreCG	3.5	Anxiety
	Right AMYG	5	Anxiety
20	Right PreCG	4	Happiness
	Right PreCG	5	Happiness
21	Left PCUN	0.5	Happiness
	Left PCUN	1	Happiness
	Left PCL	0.5	Happiness
	Left PCL	1	Happiness
22	Left THA	3	Happiness
	Left THA	4	Happiness
	Left THA	5	Happiness
	Left PUT	4	Happiness
	Left PUT	6	Happiness
23	Left HIP	2	Happiness
	Left HIP	3	Happiness
	Left HIP	4	Happiness
	Left HIP	4.5	Happiness
	Left HIP	5	Happiness
24	Left THA	6	Happiness
25	Left SFG	1	Happiness
	Left SFG	1.5	Happiness
26	Left ACC	6	Happiness
27	Left INS	1	Happiness
	Left SMG	1	Happiness
	Left SMG	2	Happiness
28	Left HIP	1.5	Depression
	Left HIP	2	Depression
	Left HIP	3	Depression
	Left HIP	4	Depression
29	Left INS	4	Depression

#### 3.1.1 Fear

Fear was elicited by 35 contacts in 16 patients. The positive contacts lay in the left hippocampus (23.52%), right hippocampus (14.71%), right fusiform (14.71%), left fusiform (8.82%), right superior frontal gyrus (8.82%), right anterior cingulate cortex (5.88%), left middle frontal gyrus (5.88%), left superior temporal gyrus (2.94%), right superior temporal gyrus (2.94%), left insula (2.94%), right insula (2.94%), left middle temporal gyrus (2.94%), and left amygdala (2.94%).

#### 3.1.2 Anxiety

Anxiety was elicited by six contacts in three patients. The positive contacts lay in the left insula (33.33%), right amygdala (33.33%), left orbitofrontal cortex (16.67%), and right precentral gyrus (16.67%).

#### 3.1.3 Happiness

Happiness was elicited by 37 contacts in nine patients. The positive contacts lay in the left paracentral lobule (21.62%), left thalamus (13.51%), left hippocampus (13.51%), left putamen (8.11%), left precuneus (8.11%), left supramarginal gyrus (8.11%), right orbitofrontal cortex (5.40%), left anterior cingulate cortex (5.40%), left superior frontal gyrus (5.40%), right precentral gyrus (5.40%), right insula (2.70%), left insula (2.70%), right superior frontal gyrus (2.70%), and right middle frontal gyrus (2.70%).

#### 3.1.4 Depression

Depression was elicited by seven contacts in three patients. The positive contacts lay in the left hippocampus (57.14%), right insula (28.57%) and left insula (14.29%).

**FIGURE 3:**
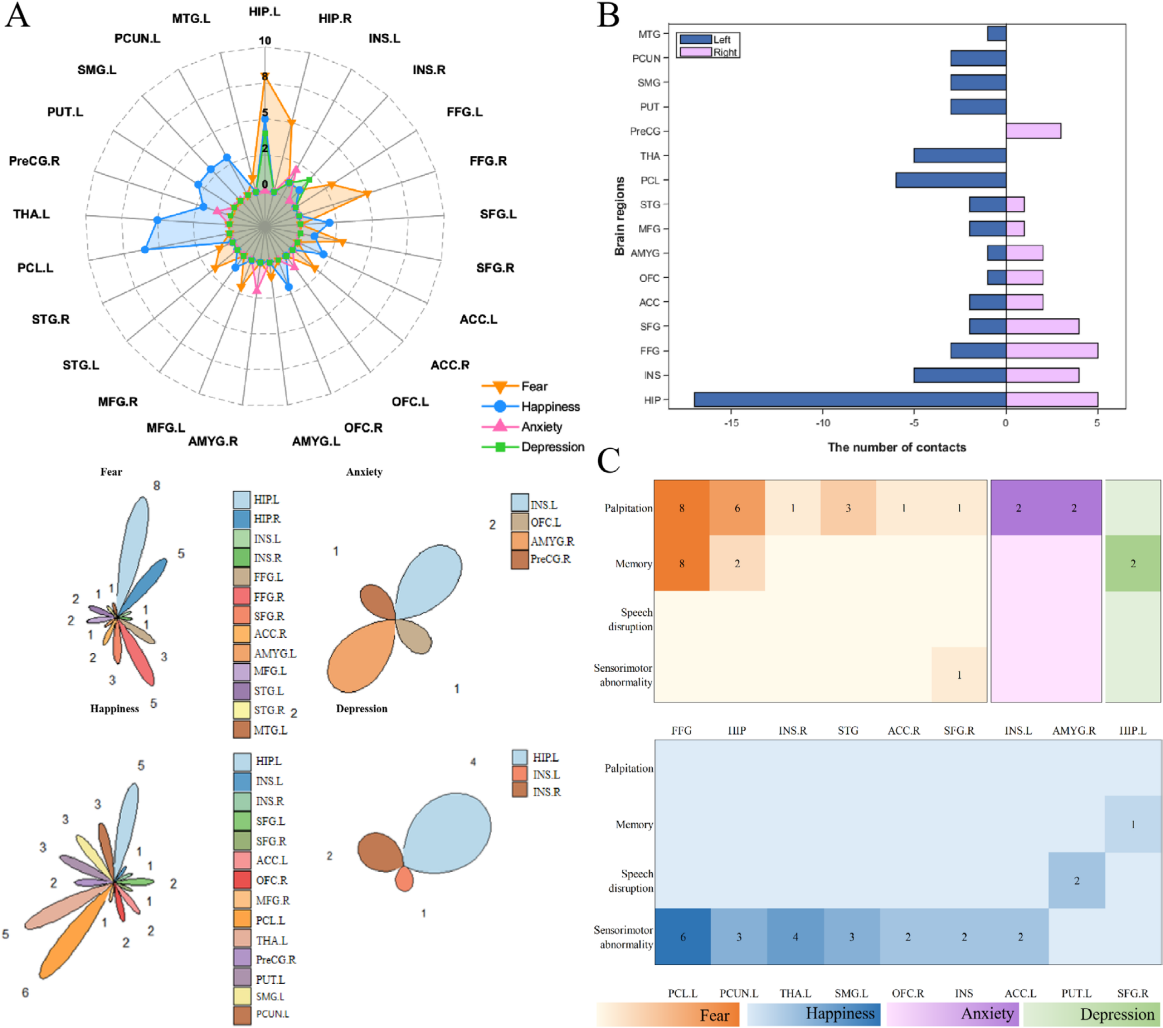
Mapping data statistics. (A) Radar and petal maps of different emotional responses and their occurrence at each direct electrical stimulation (DES) site: orange for fear, blue for happiness, purple for anxiety, and green for depression. (B) The symptoms elicited by different brain regions are reflected in different sides, obviously more symptoms appeal on the left side than the right side (C) Correlations between concomitant symptoms and contacts. The numbers represent the number of times the concomitant symptoms were observed. Darker colors indicate higher contact numbers. Colors indicate emotions: Blue for happiness, orange for fear, purple for anxiety, and green for depression. For example, “6” represents six electrode contacts eliciting concomitant symptoms during DES. In addition, “.R” represents the right side, whereas “.L” represents the left side.

Based on the statistical data provided in Table 1, three charts were generated to demonstrate the number of times each electrical cortical stimulation and emotional symptom occurred (Figure 3A,B). In addition, the concomitant symptoms elicited by the stimulus are shown in Figures 2C and 3C. Stimuli that produced only emotional symptoms are shown in Table S1 and Figure 2D.

### 3.2 Functional Connectivity

**FIGURE 4:**
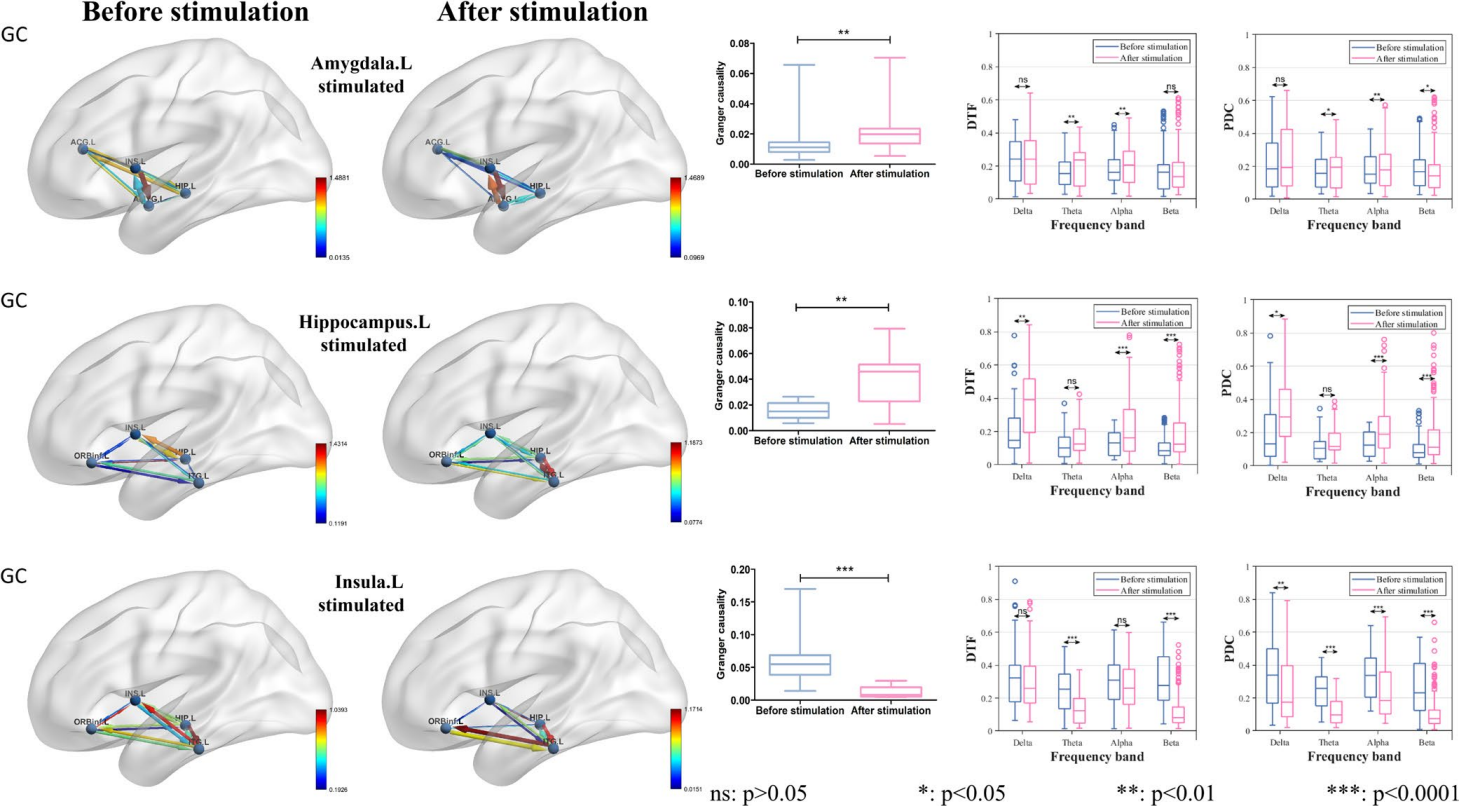
Network statistical analysis. Granger causality (GC), directed transfer function (DTF), and partial directed coherence (PDC) analyses of local networks, conducted 10 s before and after stimulation. The results revealed significant differences in GC. Moreover, variations in DTF and PDC were primarily observed within different frequency bands. It was also observed that the connectivity strength increased following stimulation of the left amygdala and left hippocampus, but decreased following stimulation of the left insula.

Next, we selectively analyzed the changes in functional connectivity for certain positive contacts of concern (the left amygdala, left hippocampus, and left insula), instead of performing functional connectivity analysis for all positive contacts. This focus was chosen because these functional changes mainly appear in the limbic system, which is closely associated with emotional responses. We aimed to determine whether the stimulus‐elicited emotional response originated from focal cortical function or was the result of a local network change. The EEG signals were preprocessed, which included channel selection focusing on four ROIs with SEEG electrode coverage. A bandpass filter was applied within the range of 0.5–100 Hz, followed by a notch filter at 50 Hz to remove line noise. The EEG data were then subjected to manual artifact rejection to eliminate noisy segments, and baseline correction was performed using a time window of −200 to 0 ms relative to stimulus onset; the sampling rate for bandpass filtering was uniformly set at 256 Hz. Finally, the time windows were defined, and EEG analysis was conducted separately for the 10‐s epochs before and after stimulus delivery. Considering individual variations in SEEG placement among patients, four specific brain regions of interest surrounding the stimulus site (with or without inclusion of the stimulus site) were analyzed. After conducting DTF and PDC analysis, we focused on statistical testing within the delta, theta, alpha, and beta frequency bands. Ultimately, our findings demonstrated that significant differences in local networks around these three ROIs elicited emotional responses. The results are presented in Figure 4, Figures S1–S3, and Table 2.

**TABLE 2:** The discrepancy in the 10‐s interval before and after stimulation was derived using various analytical approaches.

Contacts	GC	DTF	PDC
Amygdala.L	*p* = 0.0024**	*p* (Delta) = 0.5366	*p* (Delta) = 0.0854
		*p* (Theta) = 0.0071**	*p* (Theta) = 0.0140*
		*p* (Alfa) = 0.0053**	*p* (Alfa) = 0.0095**
		*p* (Beta) = 0.1408	*p* (Beta) = 0.0283*
Hippocampus.L	*p* = 0.0031**	*p* (Delta) = 0.0041**	*p* (Delta) = 0.0102*
		*p* (Theta) = 0.0695	*p* (Theta) = 0.1131
		*p* (Alfa) < 0.0001****	*p* (Alfa) < 0.0001****
		*p* (Beta) < 0.0001****	*p* (Beta) < 0.0001****
Insula.L	*p* = 0.0010**	*p* (Delta) = 0.7355	*p* (Delta) = 0.0074**
		*p* (Theta) < 0.0001****	*p* (Theta) < 0.0001****
		*p* (Alfa) = 0.2589	*p* (Alfa) = 0.0001***
		*p* (Beta) < 0.0001****	*p* (Beta) < 0.0001****

* *p* < 0.05.

** *p* < 0.01.

*** *p* < 0.001.

**** *p* < 0.0001.

Additionally, recordings from 10 s before and after each stimulus in ROIs that did not elicit an emotional response were also selected. The DTF and PDC for these durations were calculated. Subsequently, Spearman correlation analysis was performed between the DTF and PDC coefficients obtained from emotion‐neutral stimuli and those from emotionally evocative stimuli to confirm the independence of each brain electrical activity component and to eliminate potential superimposed effects. Ultimately, our findings demonstrated consistent EEG patterns across different durations in the three ROIs (*p* < 0.0001), thereby validating the significance of our previous EEG analysis. The results are presented in Figure S4.

## 4 Discussion

In this study, we generated a range of valuable findings by analyzing bulk data related to the emotional responses triggered by DES. These findings can help researchers gain a deeper understanding of the functionality of the cerebral cortex. Moreover, these data provide new research material for investigating the functionality of human emotion.

The DES method used in this study is a widely used research approach for investigating cerebral cortex functionality, particularly concerning the basic function of the cortex, and has been used for many decades [29]. This method has also been repeatedly validated in clinical practice [27]. Therefore, the emotional response triggered by DES is known to be closely related to the specific region of the brain receiving the stimulus.

Our first aim was to determine whether these positive responses were related to the patient's epilepsy network [35, 36, 37, 38]. The following three conditions were excluded from our analysis to eliminate the interference caused by the pathological basis of epilepsy: [1] electrode contacts located in the EZs, [2] electrodes producing the same emotional response as seizure auras following DES, and (3) contacts with AD after DES. Based on this, confidence was established that the positive results were closely related to human brain functionality rather than being caused by epilepsy.

In this study, we systematically delineated the brain regions associated with emotionally evoked responses through DES in the Results section. The findings revealed that key neural substrates underlying the four emotional categories predominantly localized to the limbic system, including the hippocampus, amygdala, insula, and cingulate cortex. Notably, the “happiness” response exhibited additional significant activation clusters within the neocortical regions of the frontal lobe and basal ganglia. These spatial distributions align with previous fMRI studies on emotional processing, collectively indicating that both isolated cortical functions and local network dynamics within the limbic system, frontal lobe, and adjacent regions play crucial roles in emotional elicitation. Therefore, in future studies, concurrent fMRI evidence could be integrated with DES‐mediated modulation of patients' emotional states to further validate the critical role of specific brain regions or local networks in coordinating emotional responses.

The next key issue to address was whether the emotional response elicited by DES reflected the functionality of the focal cortex itself or the activation of a localized network [1]. Similar to the previous controversy relating to emotional locationism and psychological constructivism, the locationism hypothesis holds that different emotional categories correspond to distinct states with inherent motivational characteristics that drive cognition and behavior [1]. It is assumed that these states are biologically basic and inherited, and cannot be broken down into psychological components that are more basic [1, 39]. Conversely, the constructivism hypothesis holds that the entire process of emotional response can be broken down into several parts and is related to the changes in functional connectivity of the brain [1, 8]. Therefore, we used GC analysis, DTF, and PDC to investigate the causal relationship between these positive contacts and the surrounding brain regions before and after stimulation. Our analysis revealed statistically significant changes in some network connections before and after DES. These findings suggested that the emotional responses elicited by stimuli might arise from the activation of local networks. However, we do not necessarily need to concur with the traditional orientationist and constructivist hypotheses. It appears that simple emotion may arise from changes within small networks, whereas constructivism refers to conceptualizing various external inputs in the brain through personal experience to generate emotions [1]. Combining locationism and constructivism, for example, can lead to different emotional conceptualization processes in various task states; this can result in activating some nodes in the default mode network [40]. Also, the central emotion generated after conceptualization, which is a single emotion, may be caused by the changes in the cortex or network.

Furthermore, the study revealed that DES of the amygdala and hippocampus led to enhanced functional connectivity within local networks post‐stimulation, whereas stimulation of the insula resulted in reduced local network connectivity. These opposing experimental outcomes suggest that distinct brain regions occupy different hierarchical positions within local networks. Specifically, the amygdala and hippocampus may function as higher‐order regions, facilitating top‐down signal propagation and thereby strengthening local network connectivity upon stimulation. In contrast, the insula appears to operate within a downstream system, where stimulation induces feedback inhibition, attenuating signal transmission within the network.

Next, we attempted to determine whether the emotional responses elicited by DES were related to concomitant physical symptoms. The existence of emotional responses with concomitant physical symptoms is debatable in the field of emotion research. Our findings showed that some symptoms were accompanied by somatic or visceral symptoms, whereas others were not. We believe that the more physical symptoms that were evident, the more the evidence revealed that DES activated the functional connections in the corresponding brain regions, rather than a focal cortical function [23, 27].

Although our study does not completely address the questions posed earlier, it provides a novel perspective on emotional problems as well as an objective method for eliciting emotional responses. We hope that our findings provide a valuable reference for developing new research strategies in the future. For instance, in future studies, dynamic causal modeling (DCM) could be integrated to enhance the robustness of the data, thereby providing more precise delineation of the local networks underlying emotional responses. Ultimately, by modulating these local networks, a closed‐loop emotion regulation framework could be established, laying the groundwork for subsequent therapeutic intervention in psychiatric disorders.

## 5 Limitations

The number of electrode contacts implanted in each brain area depends on the location of the patient's epilepsy focus and, hence, is inconsistent across all patients. Therefore, the percentage of stimulation in a specific brain area to elicit an emotional response cannot be considered absolutely authoritative. Hence, a larger number of SEEG implantation cases in different brain areas need to be considered. In addition, we excluded affective responses similar to the ictal aura and the electrode contacts located in the Seizure onset zone (SOZ) or eliciting AD to investigate transient emotional symptoms in normal brain regions. However, these strict inclusion criteria might have led to the omission of key results. Moreover, some epileptic foci receiving stimulation might have led to different changes in functional brain connections; therefore, our findings may not be fully representative of normal human brain function. In future research, non‐invasive techniques such as transcranial magnetic stimulation (TMS) could also be employed to modulate emotional states in healthy individuals. Extending this methodological approach to normal populations would represent a significant breakthrough. Furthermore, only an exemplary analysis of positive contacts in a small number of patients was performed to preliminarily verify our hypothesis. Finally, due to the variability in inter‐stimulus intervals, a rigorous screening process was conducted, and the 10‐s epochs before and after stimulation were identified as the most complete datasets. In future studies, the duration of pre‐ and post‐stimulation periods will be extended to further enhance the precision of the results.

## 6 Conclusions

We analyzed a large body of DES‐elicited emotional response data to provide a new perspective for investigative research and an objective method with which to elicit emotional responses, contrary to previous investigations. We hope that our data will facilitate further research in the field of emotion.

## Author Contributions

Tao Yu conceived and supervised the study. Xiaoming Yan, Xiaohua Zhang, Xueyuan Wang, and Wei Shu recruited patients and performed experiments. Menglin Liu, Ying Gao, and Guiliang Hao organized and analyzed data. Menglin Liu plotted graphics with Guiliang Hao and Ying Gao. Menglin Liu, Ying Gao, and Tao Yu wrote the manuscript. Menglin Liu, Xiaoming Yan, and Tao Yu made manuscript revisions. All authors reviewed the results and approved the final version of the manuscript.

## Ethics Statement

This study was approved by the Medical Ethics Committee of Xuanwu Hospital, Capital Medical University (Approval reference: LYS [2024] 297‐001). The foundation of the study lies in the retrospective collection of clinical data, which is not a prerequisite for future research endeavors. Furthermore, it has been explicitly stated in the ethical approval documentation that individual informed consent from patients was waived for our investigation. Hence, we obtained universal informed consent from all patients.

## Consent

All patients provided informed consent after admission, and legal guardians provided consent for underage participants.

## Conflicts of Interest

The authors declare no conflicts of interest.

## Supporting information


Data S1.

## Data Availability

The data that support the findings of this study are available from the corresponding author upon reasonable request.
