# Supplementary material for: Symptomatic Emotional Responses and Changes in Networks Elicited by Direct Electrical Stimulation
Source: CNS Neurosci Ther. 2025 Apr 17;31(4):e70393. doi: 10.1111/cns.70393 (PMC12004395; doi:10.1111/cns.70393)
Supplement: Supplementary file 1 — Data S1. [file CNS-31-e70393-s001.pdf]

eMethod. Explanation of Directed Transfer Function (DTF) and Partial Directed Coherence (PDC).

eTable. Demographic and clinical profiles of the subjects without concomitant symptoms.

eFigure 1. directed transfer function (DTF), and partial directed coherence (PDC) before and after stimulation of the left amygdala.

eFigure 2. directed transfer function (DTF), and partial directed coherence (PDC) before and after stimulation of the left hippocampus.

eFigure 3. directed transfer function (DTF), and partial directed coherence (PDC) before and after stimulation of the left insula.

eFigure 4. The DTF and PDC coefficients of 10s before and after the stimulus (20s totally) were subjected to Spearman correlation analysis.

This supplementary material has been provided by the authors to give readers additional information about their work.

eMethods. Explanation of Directed Transfer Function (DTF) and Partial Directed Coherence (PDC)

The estimation of Directed Transfer Function (DTF) is conducted using a multivariable autoregressive (MVAR) model, facilitating the identification of directed connections between brain structures. DTF computation involves the utilization of the inverse matrix of the coefficient matrix in the model, enabling the capture of both direct and indirect interactions among different brain regions. However, one limitation associated with DTF is its potential to detect false connection information due to indirect causal links. On the other hand, Partial Directed Coherence (PDC) possesses the capability to quantify direct flow of information from one channel to another at a specific frequency while minimizing interference effects from other channels. PDC values range between [0, 1], with larger values indicating stronger flow of information from one channel to another. The advantage lies in PDC's ability to provide direct structural insights into directed connections within multivariable time series data; however, it is sensitive to noise and assumes linearity in data.

eTable. Demographic and clinical profiles of the subjects without concomitant symptoms

Patient No.	Patient information	Handed-ness	Emotional responses	Locations
1	Juvenile	Right	Fear	Left insula/Left hippocampus
2	Adult	Right	Fear	Right superior anterior cingulate
3	Juvenile	Right	Fear	Left amygdala
4	Adult	Right	Fear	Right hippocampus
5	Adult	Right	Fear/Happiness	Right superior frontal cortex/Right middle frontal
6	Adult	Right	Anxiety	Left orbitofrontal cortex
7	Juvenile	Right	Anxiety	Right anterior central cortex
8	Adult	Right	Happiness	Left putamen
9	Adult	Right	Happiness	Left dorsal thalamus
10	Juvenile	Right	Depression	Right insula
11	Adult	Right	Depression	Left hippocampus
12	Adult	Right	Depression	Left insula

eFigure 1. directed transfer function (DTF), and partial directed coherence (PDC) before and after stimulation of the left amygdala

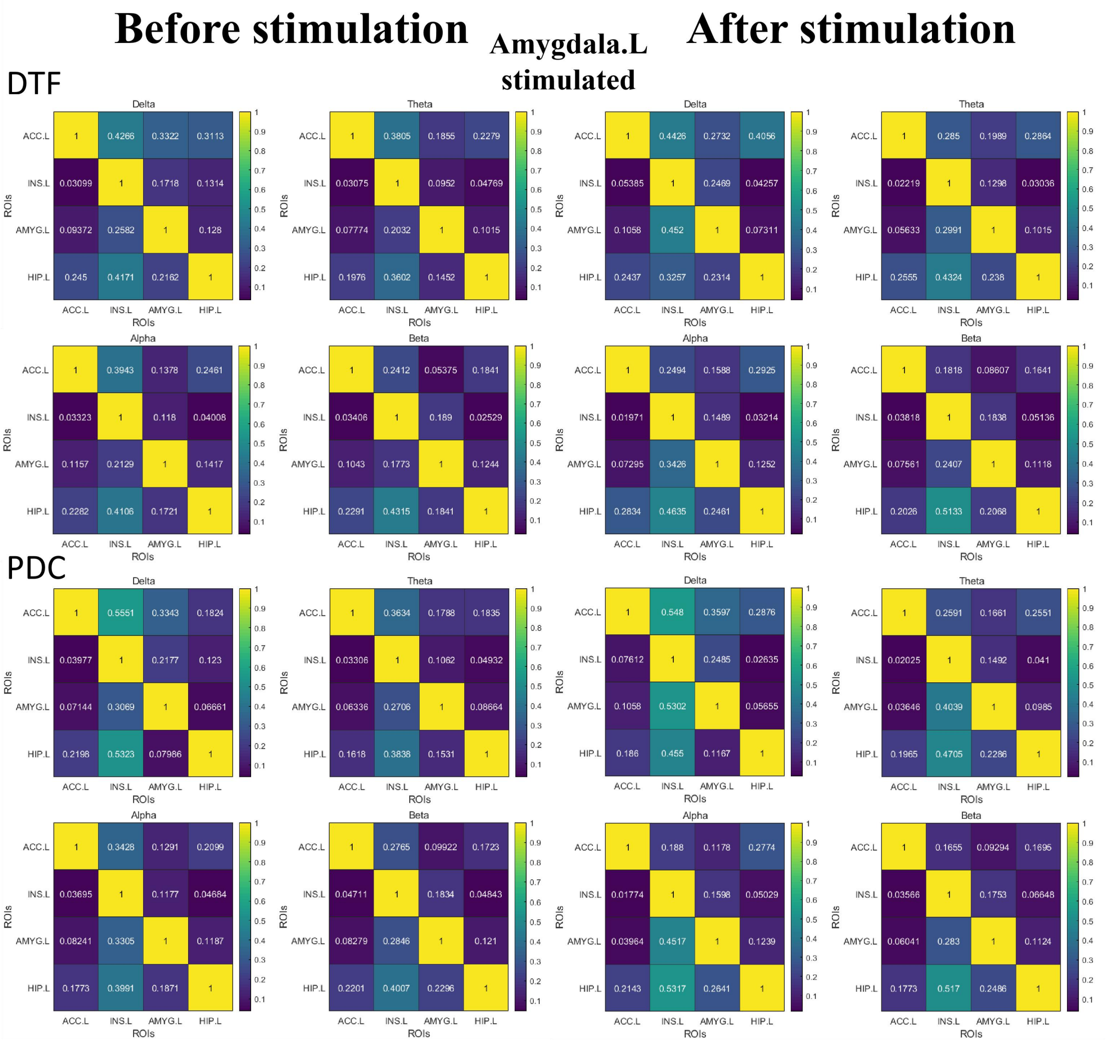

After the induction of depression through left amygdala stimulation, the directed transfer function (DTF), and partial directed coherence (PDC) of the left anterior cingulate cortex (ACC.L), left insula (INS.L), left amygdala (AMYG.L), and left hippocampus (HIP.L) were analyzed for a duration of 10 seconds before and after stimulation. Notably, it was observed that the connectivity strength increased following stimulation.

eFigure 2. directed transfer function (DTF), and partial directed coherence (PDC) before and after stimulation of the left hippocampus

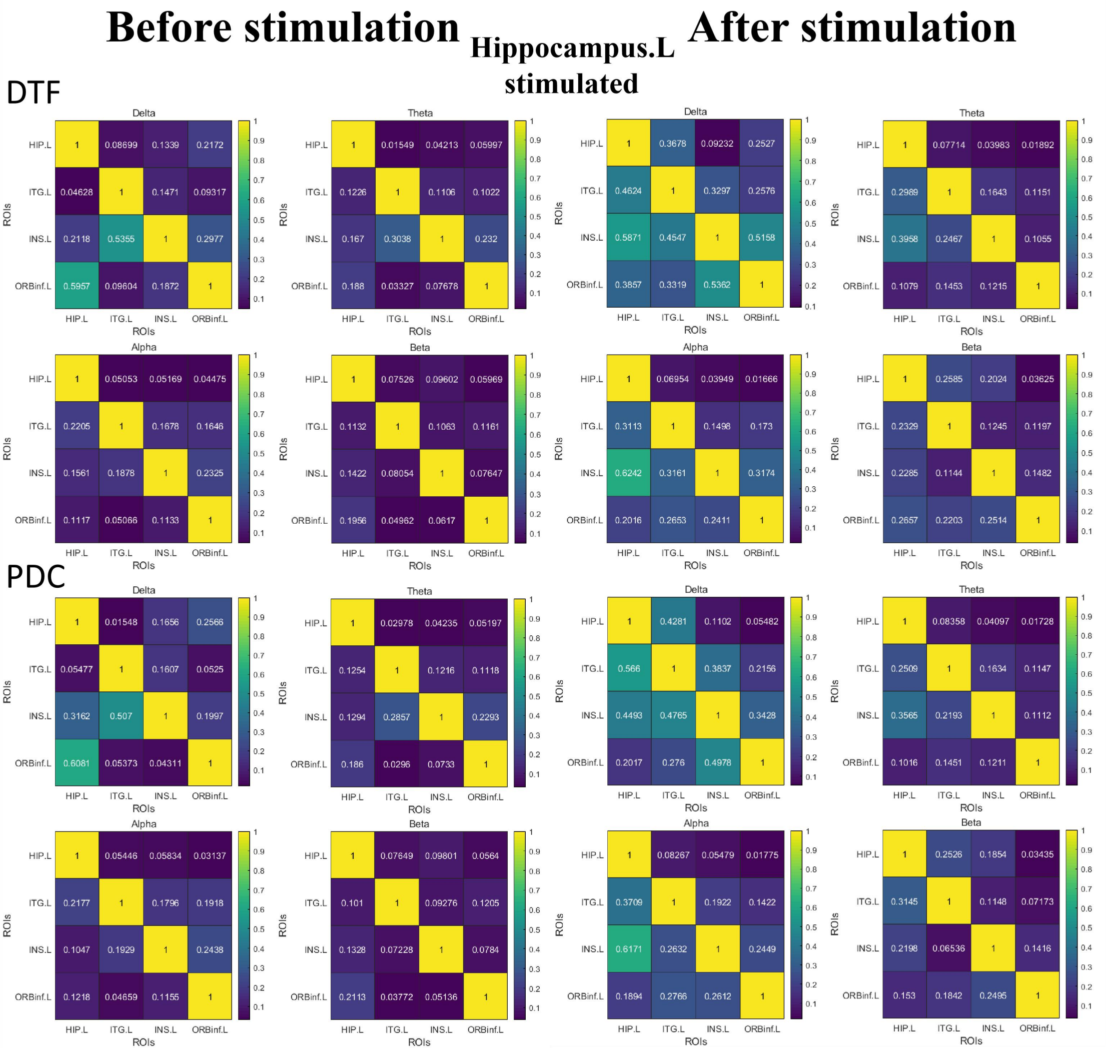

After the induction of fear through left hippocampus stimulation, the directed transfer function (DTF), and partial directed coherence (PDC) of the left hippocampus (HIP.L), left inferior temporal gyrus (ITG.L), left insula (INS.L), and left inferior Orbitofrontal cortex (ORBinf.L) were analyzed for a duration of 10 seconds before and after stimulation. Notably, it was observed that the connectivity strength increased following stimulation.

eFigure 3. directed transfer function (DTF), and partial directed coherence (PDC) before and after stimulation of the left insula

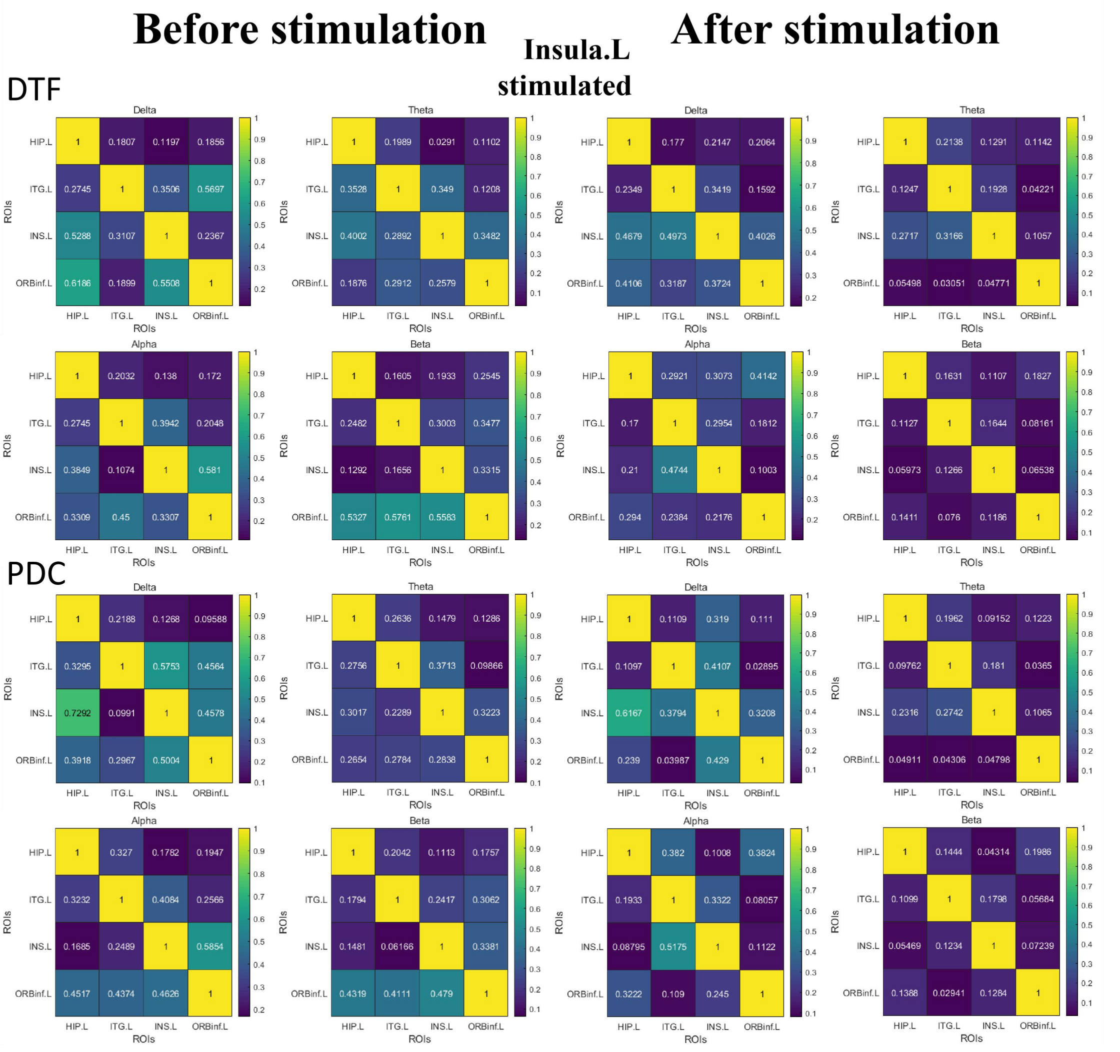

After the induction of fear through left insula stimulation, the directed transfer function (DTF), and partial directed coherence (PDC) of the left hippocampus (HIP.L), left inferior temporal gyrus (ITG.L), left insula (INS.L), and left inferior Orbitofrontal cortex (ORBinf.L) were analyzed for a duration of 10 seconds before and after stimulation. Notably, it was observed that the connectivity strength decreased following stimulation.

eFigure 4. The DTF and PDC coefficients of 10s before and after the stimulus (20s totally) were subjected to Spearman correlation analysis.

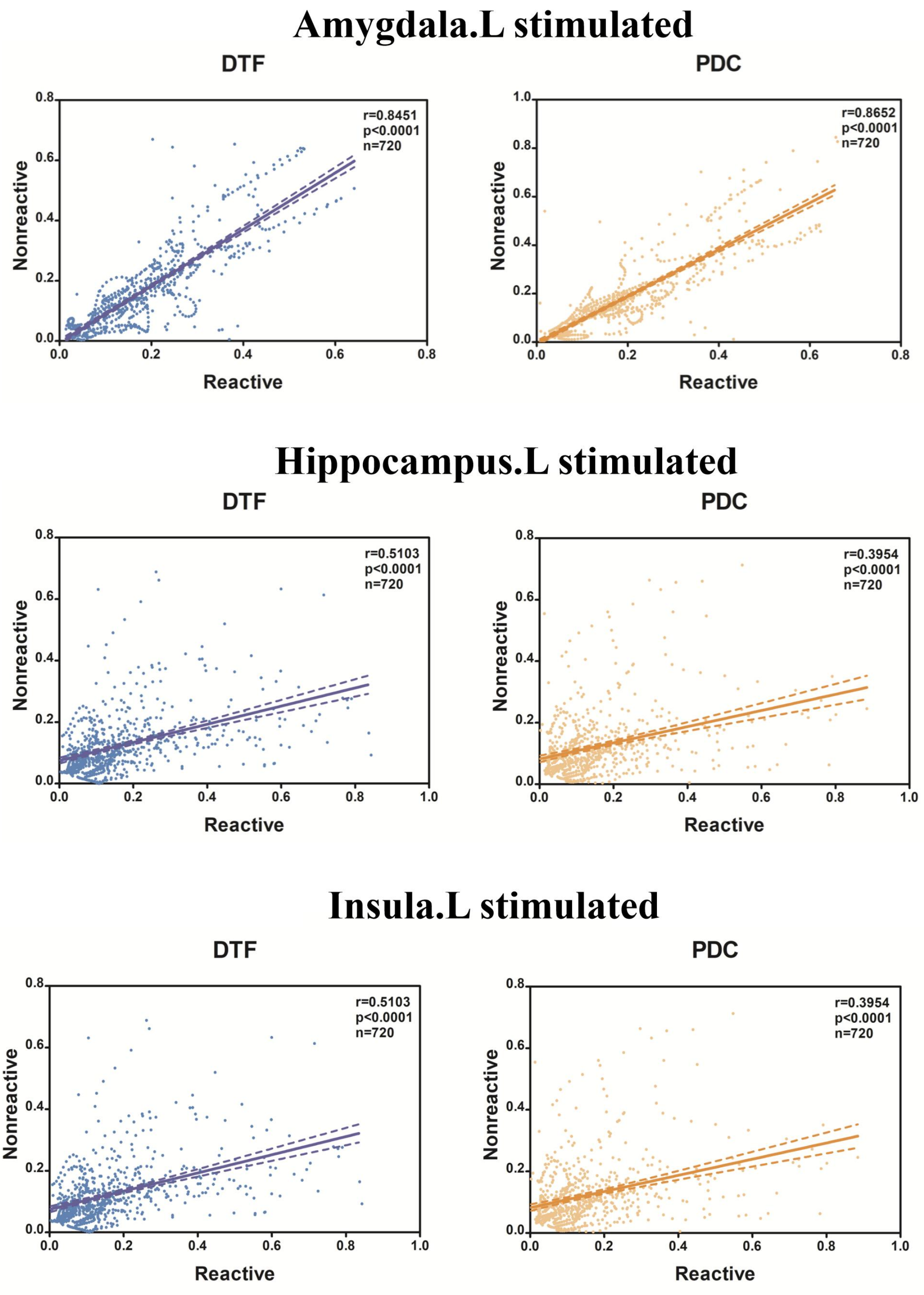

It was observed that across all regions of interest (ROIs), the electrophysiological

coefficients associated with emotional stimuli exhibited consistency with those of non-emotional stimuli ($p<0.0001$).
